# Decay in Retinoic Acid Signaling in Varied Models of Alzheimer’s Disease and *In-Vitro* Test of Novel Retinoic Acid Receptor Ligands (RAR-Ms) to Regulate Protective Genes

**DOI:** 10.3233/JAD-190931

**Published:** 2020-02-04

**Authors:** Thabat Khatib, David R. Chisholm, Andrew Whiting, Bettina Platt, Peter McCaffery

**Affiliations:** a Institute of Medical Sciences, University of Aberdeen, Foresterhill, Aberdeen, Scotland, UK; b Department of Chemistry, Durham University, Science Laboratories, South Road, Durham, UK

**Keywords:** Aldh1a2, amyloid, chemokines, cholesterol, cyp26a1, cyp26b1, growth factor, Nos2, stra6, Tnf

## Abstract

Retinoic acid has been previously proposed in the treatment of Alzheimer’s disease (AD). Here, five transgenic mouse models expressing AD and frontotemporal dementia risk genes (i.e., PLB2_APP_, PLB2_TAU_, PLB1_Double_, PLB1_Triple_, and PLB4) were used to investigate if consistent alterations exist in multiple elements of the retinoic acid signaling pathway in these models. Many steps of the retinoic acid signaling pathway including binding proteins and metabolic enzymes decline, while the previously reported increase in RBP4 was only consistent at late (6 months) but not early (3 month) ages. The retinoic acid receptors were exceptional in their consistent decline in mRNA and protein with transcript decline of retinoic acid receptors β and *γ* by 3 months, before significant pathology, suggesting involvement in early stages of disease. Decline in RBP1 transcript may also be an early but not late marker of disease. The decline in the retinoic acid signaling system may therefore be a therapeutic target for AD and frontotemporal dementia. Thus, novel stable retinoic acid receptor modulators (RAR-Ms) activating multiple genomic and non-genomic pathways were probed for therapeutic control of gene expression in rat primary hippocampal and cortical cultures. RAR-Ms promoted the non-amyloidogenic pathway, repressed lipopolysaccharide induced inflammatory genes and induced genes with neurotrophic action. RAR-Ms had diverse effects on gene expression allowing particular RAR-Ms to be selected for maximal therapeutic effect. Overall the results demonstrated the early decline of retinoic acid signaling in AD and frontotemporal dementia models and the activity of stable and potent alternatives to retinoic acid as potential therapeutics.

## INTRODUCTION

Alzheimer’s disease (AD) is a chronic multifactorial neurodegenerative disorder that is characterized by the accumulation of amyloid plaques and neurofibrillary tangles, progressive memory impairment, and the deterioration of cognitive ability [1]. It is the most common cause of dementia and contributes to about 60% to 70% of all cases [2]. Today, approximately 47 million people have AD worldwide, and the number is expected to reach more than 131 million by 2050 [3].

Retinoic acid (RA), the active metabolite of vitamin A, is well studied from its role in development of the central nervous system (CNS), but more recently evidence has accumulated on the importance of RA signaling in the adult CNS [4]. The components of the RA signaling system are present in the adult brain such as the synthetic and catabolic enzymes, receptors, and binding proteins, although the distribution pattern differs from the one observed in the embryonic CNS [5]. RA is known to be crucial for several aspects of neuroplasticity within the adult brain. Neuroplasticity is fundamental to the formation of new memories [6], and aspects of neuroplasticity promoted by RA are long-term potentiation/depression (LTP/LTD) [7], neurogenesis [8], homeostatic plasticity [9], and the capacity to form new neurites and neurite extensions [10].

Use of RA for AD is suggested by findings such as low endogenous retinol levels correlating with cognitive decline in the aging human [11] and that RA falls in the aging rodent brain [12, 13]. Mingaud et al. have shown that age-related downregulation of RA signaling disrupts hippocampal LTP and cellular properties, and that retinol supplementation reverses the effects [13]. Among the first proposal of a link between retinoids and AD, Goodman and Pardee reported a genetic linkage between AD and RA using the Locus Link database, that showed a large number of genes implicated in AD pathology are located at chromosomal loci together with RA signaling system encoding genes [14]. In addition, several studies reported a decline in RA signaling in various AD models [15].

As RA supports neuronal survival and neuroplasticity, essential for learning and memory, this decline weakens cognitive function [4]. If decreased RA levels promote AD, it would be anticipated that restoration of levels would be protective and several *in vitro* studies have indicated that RA reduces amyloid-β (Aβ) neurotoxicity [16, 17]. Furthermore, it was shown that a vitamin A-deficient diet in rodents leads to disruption in the RA signaling system and Aβ deposition in the cerebral blood vessels of forebrain neurons, and that these changes were reversed by RA administration [18, 19]. RA also inhibits the production of different cytokines and chemokines, such as interleukin 6 [20, 21], involved in the inflammatory response of many age related diseases. For example, the mRNA levels of interleukin 6 increase early in the hippocampus and cortex of Tg2576 AD model mice [22]. RA also inhibits many aspects of microglia activation, such as tumor necrosis factor alpha production and the expression of inducible nitric oxide synthase [23]. Such anti-inflammatory actions of RA will be beneficial for treatment of neurodegenerative disease.

Boosting the RA signal with synthetic ligands for its receptor improves cognition in transgenic mouse models of AD, clearing Aβ in both neurons and microglia as well as providing a strong anti-inflammatory action [24]. Hence, synthetic retinoids may provide a treatment for AD and other neurodegenerative disorders. Tamibarotene (Am80) is an example of a synthetic retinoid that is studied extensively as a candidate drug for AD because of its various beneficial effects. Kawahara et al. reported that administration of Am80 decreased the level of insoluble Aβ_42_ in APP23 AD model mice [25]. Am80 neuroprotective effects were also observed in inflammation-induced midbrain neurons by increasing brain-derived neurotrophic factor levels [26]. Acitretin is another retinoid drug currently studied. Acitretin was reported to increase the levels of the *α*-secretase (ADAM10) of amyloid-β protein precursor (AβPP), driving the non-amyloidogenic pathway in neuroblastoma cells with reduction in Aβ levels in APP/PS-1 AD model mice [27]. In addition, Acitretin was reported to cross the blood-brain barrier (BBB) in mice [28]. Endres et al. investigated the changes in *α*-secretase-derived AβPP (AβPPs-*α*) levels in the cerebrospinal fluid (CSF) of mild to moderate AD patients after oral acitretin therapy. Results showed that acitretin increased AβPPs-*α* levels and enhanced non-amyloidogenic AβPP processing in human patients [29].

A significant problem with the study of dementia/AD models is that most are only a model of a single hypothesis for the cause of AD. A comprehensive understanding of the disease is necessary to develop successful therapeutics that will tackle the majority of cases. This study used multiple, genetically comparable transgenic knock-in mouse models of AD, and models of tau pathology associated with AD and frontotemporal dementia (FTD), to investigate alterations in RA signaling at the gene and/or protein level in these models. Hippocampal and cortical mixed primary cultures from Sprague Dawley rats were used as well to perform an initial test of the therapeutic potential of a group of novel synthetic retinoids (RAR-Ms) active with genomic and non-genomic targets [30]. The capacity of these RAR-Ms to beneficially activate or repress Aβ processing genes and anti-inflammatory/neuroprotective genes, in primary neuron/glia cultures suggests retinoids as a line of research of high potential for AD treatment.

## METHODS

### Retinoid solutions

**Fig.1:**
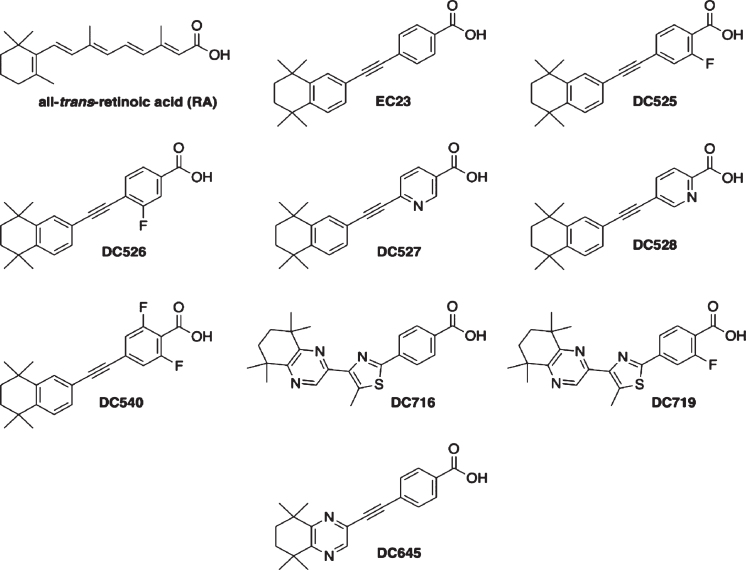
Molecular structures of the RAR-Ms used in the study.

All-*trans*-RA (Sigma-Aldrich) was dissolved at 0.1 M in dimethyl sulfoxide (DMSO) under red light, aliquoted and stored under N_2_ at –70 °C, protected from light. Synthetic retinoids (RAR-Ms) were dissolved in DMSO to give 0.01 M stock solutions. RAR-Ms were designed and synthesized as described previously [31–37]. The molecular structures of the RAR-Ms are shown in Fig. 1. The majority of the compounds exhibit high affinity for the RA receptors (RARs).

### Transgenic animal models

Five types of transgenic knock-in mouse models of in-house developed knock-in (PLB) mouse lines were studied to determine if there are any defects in the RA signaling system at the gene and/or protein levels. The PLB1_Double_ mouse model expresses human mutated APP and Tau (human APP Swedish and London mutations: K670N, M671L and V717I; human Tau mutations: P301L and R406W). PLB1_Triple_ expresses human mutated APP, Tau, and PS1 and was created by crossing the PLB1_Double_ model with an existing presenilin (PS1) mice (A246E) to get the three-gene combination [38]. The two monogenic mouse models APP (PLB2_APP_) [39] and Tau (PLB2_TAU_) were created by crossing the double transgenic mouse model PLB1_Double_ with deleter mice (Flp or Cre expressing mice that excise the FRT flanked or floxed regions respectively) [40]. The fifth model, PLB4, expresses human BACE1 [41]. The wild type (WT) control animals (PLB_WT_) used in the study were generated out of the parental PLB1_Double_, strain, followed by multiple generation crossing with C57BL/6 (Harlan Laboratories Ltd) from human APP and Tau negative littermates. All PLB mice were maintained on the same C57BL/6 background.

The PLB_WT_ (*n* = 24 males and 4 females), PLB2_APP_ (*n* = 5 males and 1 female), PLB2_TAU_ (*n* = 8 males), PLB1_Double_ (*n* = 7 males and 5 females), PLB1_Triple_ (*n* = 5 males), and PLB4 (*n* = 6 males) mice were killed at 6 months of age by cervical dislocation, while PLB_WT_ (*n* = 12 males), PLB2_APP_ (*n* = 4 males), PLB2_TAU_ (*n* = 12 males), and PLB1_Double_ (*n* = 7 males) mice were killed at 3 months age. The brains were removed, snap frozen in liquid nitrogen, weighed, and stored in microtubes at –70°C until needed. Each frozen brain was cut along the sagittal plane into two halves and one half was used for ribonucleic acids (RNA) extraction and quantitative polymerase chain reaction (qPCR) analysis work while the other half was used for protein sample preparation and western blotting analysis. Different ratios of males and females were used in the study of different mouse models which might differentially influence the results. There is little published on the influence of gender on RA signaling and so for two different AD models, a wide variety of retinoid signaling genes in the brain were compared between genders. There was no significant difference between males and females (Supplementary Figure 1).

Animals were bred at Charles River UK and delivered to the University of Aberdeen animal facility at least one week before use. All animals were housed and tested in accordance with UK Home Office, the EU directive 63/2010E and the Animal (Scientific Procedures) Act 1986.

### Rat primary cultures

Sprague Dawley (SD) rat pups were used to prepare primary neuron cultures. Rat primary cultures were used because hippocampal neurons are more numerous and faster maturing in rats than mice [42]. The rat pups were killed at postnatal day zero or one (P0-P1) by cervical dislocation and the heads were removed and placed on ice until dissection. The brains were dissected within 1 h of cervical dislocation.

6-Well plates were coated with 1 ml of poly-L-lysine (PLL; Sigma-Aldrich) solution at a final concentration of 0.002%. The plates were incubated for at least 2 h at 37°C. Two thorough washes with sterile PBS followed and afterwards the plates were air dried in the cell culture hood and stored at 4 °C in the fridge wrapped in parafilm until use.

The cortex and hippocampus from rat pup brains were rapidly dissected on a sterile 35 mm culture dish containing cold serum-free Neurobasal medium (Thermo Fisher Scientific) within 1 h of the cervical dislocation procedure. The collected tissues were placed inside Bijou containers with Neurobasal medium on ice. Following dissection as much Neurobasal medium as possible was removed leaving the tissue at the bottom of the containers. Two ml of 0.05% trypsin-EDTA solution (Thermo Fisher Scientific) were then added and the tubes were incubated at 37 °C for 15 min. The trypsin was then removed and replaced with the same volume of 1 mg/ml soybeans trypsin inhibitor (Thermo Fisher Scientific) and the container incubated for 5 min at room temperature. Afterwards, trypsin inhibitor was removed and 3 ml of supplemented Neurobasal medium with 1% Glutamax (Thermo Fisher Scientific), 2% B27 without vitamin A (Thermo Fisher Scientific), and 1% penicillin-streptomycin (Thermo Fisher Scientific) were added above the tissue of each of the tubes. The tissue was gently triturated using a fire polished glass Pasteur pipette and the cloudy medium containing the dissociated cells was passed through a 40 μm cell sieve into 50 ml falcon collection tubes. The process was repeated until the tissue was dissociated completely. The cells were then centrifuged at a relative centrifugal force (rcf) of 226 for 4 min at room temperature using a Mistral 3000i centrifuge (MSE) and resuspended in 2 ml of fresh supplemented Neurobasal medium. Cells were counted using the TC10^TM^ automated cell counter instrument (Bio-Rad) by mixing 15 μl of 0.4% trypan blue with 15 μl of cell suspension in 0.6 ml microtube and then adding 2×10 μl of the mix to the dual chamber cell counting slides (Bio-Rad). After that, 30,000 cells were plated per well and the media was changed every 2 days. The cells were used in experiments after 14 days *in vitro* (DIV).

### Retinoid treatment of primary cultures

To examine the influence of RA and RAR-Ms on the expression of a group of genes involved in AD, the cells in wells were treated with RAR-Ms for 24 h in triplicate. 24 h was chosen as the optimum treatment time from a preliminary study comparing 6 and 24 h (Supplementary Figure 2). Each experiment was repeated three times. RNA was then extracted from treated cells for qPCR analysis. To examine the influence of RA and other synthetic RAR-Ms on inflammation, the cells were treated first with 1 μg/ml lipopolysaccharide (LPS; Sigma-Aldrich) for 6 h to induce inflammation followed by RA/RAR-Ms treatment for 24 h. Subsequently, RNA was extracted from the treated cells for qPCR analysis.

### Gene expression analysis

Total RNA was extracted from primary cultures treated with 10 nM RAR-M for 24 h or frozen half brain tissues of transgenic AD knock-in mouse models using a Qiagen RNeasy mini kit according to the manufacturer’s protocol. cDNA was synthesized from 250 ng total RNA from cells or 500 ng total RNA from tissues using High qScript cDNA Synthesis master mix. qPCR reactions using PerfeCTa SYBR Green SuperMix were performed on a Roche LightCycler 480 and analyzed using LightCycler 480 1.5 software. Primers were designed using Primer-BLAST [43]. GenNorm primer kits were used for housekeeping genes (PrimerDesign). Standard curves and blank controls were run for all sets of primers tested. A housekeeping gene expression experiment was carried out for each model, and the data obtained were analyzed using the free RefFinder software [44] to determine the best reference genes for each experiment. RNA levels of target genes in PLB models and rat cultures were normalized to the appropriate reference RNA levels according to each experiment and compared to levels in wild type mouse model (PLB_WT_) or control untreated cells (CT) which were set at 1. In 6-month-old PLB models experiments, *Gapdh* and *B2m* reference genes were used with PLB1_Triple_, *Nono* and *Ywhaz* were used with PLB2_APP_ and PLB2_TAU_, *Gapdh* and *Ywhaz* with PLB1_Double_, and *Gapdh* and *Rpl13a* with PLB4. In 3-month PLB models experiments, *Ywhaz* and *Rpl13a* were used with PLB2_APP_, PLB2_TAU_, and PLB1_Double_. *Actb* reference gene was used in rat cultures experiments. The sequence of primers used is shown in Table 1.

**Table 1:** Primer sequences for genes used in qPCR analysis

		Primer Sequences	
Gene	Species	Forward Primer (5’ ⟶ 3’)	Reverse Primer (5’ ⟶ 3’)
Abca1	Rat	TGTGGAATCGTCCCTCAGTT	CATCGATGGTCAGCGTGTCA
Abcg1	Rat	GGCCGCTTTCTCGGTCG	TTCAGGTGCCCATTAAGCAGAT
Actb	Rat	CCACACCCGCCACCAGTTCG	TACAGCCCGGGGAGCATCGT
Apoe	Rat	TCCCCTGCTCAGACCCC	GTCACCTCCAGCTCTCCCT
App	Rat	GTTGAGCCTGTCGACGC	AAGCCTGAATCATGTCCGAACT
Bace1	Rat	CCGGCGGGAGTGGTATTATG	CTTGGGCAAACGAAGGTTGG
Ccl5	Rat	CCATATGGCTCGGACACCAC	GCGGTTCCTTCGAGTGACAA
Ccl3	Rat	TCCTATGGACGGCAAATTCCA	CAGATCTGCCGGTTTCTCTTG
Crabp1	Mouse	GGCGTTGGGTGTGAACGCCA	GGCCAGCTCTCGGGTCCAGT
Crabp2	Mouse	GATGGAGAGCTGATCCTGACAATGAC	TGCTGACCTGGGAGGGGCAA
Rbp1	Mouse	GCAAGTGCATGACCACTGTGAGC	TTCGCTGGCAGAAGCCTGGG
Cyp26a1	Mouse	GCCTGTACCGGGGCGTGAAG	GTGACGTCGCAGCACTGGCT
Cyp26b1	Mouse	CCAGGACTGTATGCCCATGA	CCACTCACCAACAAAAAGACAAG
Ide	Rat	TAGCAGGCCTGAGCTATGATCT	TGGCTGTTTGTCATTGTAACCT
Igf1	Rat	GAAGCGATGGGGAAAATCAGCA	CGAGCTGGTAAAGGTGAGCAA
Igf2	Rat	GGAGGGGAGCTTGTTGACAC	TATGTCTCCAGGAGGGCCAA
Il1b	Rat	GTGCAAGTGTCTGAAGCAGC	CCCAAGTCAAGGGCTTGGAA
Il12a	Rat	GCCTGCTTACCACTGGAACT	CCAAGGCACAGGGTCATCAT
Adam10	Rat	CAAACGAGCAGTCTCACACG	TGTCCCTCTTCATTCGTAGGT
Mme	Rat	TTGCACCGGGGTTCATTTG	TTCTTCGGCTTTGGAGCATTG
Nos2	Rat	GGACTTTTAGAGACGCTTCTGAG	CTCTGAAGAGAAACTTCCAGGGG
Aldh1a2	Mouse	CAAGGAGGCTGGCTTTCCACCC	GGGCTCTTCCCTCCGAGTTCCA
Aldh1a3	Mouse	TCAAAGAGGTCGGGTTCCCTCCG	AGGCGGCTTCTCTGACCAGCT
Rar*α*	Mouse	CGCCAAGGGAGCTGAACGGG	GGGTGGCTGGGCTGCTTCTG
Rarβ	Mouse	ACACCACGAATTCCAGCGCTGAC	CAGACCTGTGAAGCCCGGCA
Rar*γ*	Mouse	CCTGTGAAGGCTGCAAGGGCT	GTCGGGCGAGCCCTCCTCTT
Rbp4	Mouse	ACGAGTCCGTCTTCTGAGCAACTG	GCACAGCTCCTCCTGCCGTT
Sod1	Rat	AGGATTAACTGAAGGCGAGCA	GGTCTCCAACATGCCTCTCTT
Stra6	Mouse	TTGTGCTTCGGCAGGGCACC	CTGGTCTGCAGCCCCTGGGA
Tnf	Rat	GATCGGTCCCAACAAGGAGG	TTTGCTACGACGTGGGCTAC

### Protein analysis

Frozen half brain tissues of all male transgenic PLB knock-in mouse models (4 PLB_WT_, 5 PLB2_TAU_, and 5 PLB1_Double_) were homogenized mechanically in NP-40 lysis buffer containing protease inhibitor cocktail (Sigma-Aldrich, 10 μl per 1 mg) using manual disposable pestles. Protein concentrations were measured using a BCA assay kit (Thermo Fisher Scientific). 30 μg protein was loaded and separated by electrophoresis through 12% SDS-polyacrylamide gels and transferred to nitrocellulose membranes. Western blots were developed using enhanced chemiluminescence (Millipore) and the protein bands were detected and scanned using a myECL Imager (ThermoScientific). The Ponceau S staining was used as a loading control method. The levels of our proteins of interest were normalized to total protein levels rather than using a specific reference protein such as β-actin [45–49], because the housekeeping protein levels may vary depending on disease state especially in neuropathological events [50]. After normalization, target protein levels were compared to protein levels in wild type mouse models (PLB_WT_) which were set at 1.

Western blot antibodies used are shown in Table 2.

**Table 2:** List of antibodies used in western blotting

Primary Antibody	Host	Dilution	Predicted Molecular Weight	Supplier (CAT#)
CYP26A1	Rabbit	1 : 200	56 kDa	Source Bioscience (CYP26A11-A)
CYP26B1	Rabbit	1 : 500	58 kDa	Proteintech (21555-1-AP)
RALDH1	Rabbit	1 : 1000	54 kDa	Abcam (ab24343)
RALDH2	Rabbit	1 : 3000	55 kDa	Millipore (ABN420)
RALDH3	Rabbit	1 : 500	53 kDa	Millipore (ABN427)
RAR*α* (C-20)	Rabbit	1 : 1500	51 kDa	Santa Cruz (sc-551)
RARβ	Rabbit	1 : 500	50 kDa	Abcam (ab53161)
RAR*γ* (C-19)	Rabbit	1 : 200	50 kDa	Santa Cruz (sc-550)
RDH10	Rabbit	1 : 500	39 kDa	Proteintech (14644-1-AP)
STRA6 (Q-14)	Goat	1 : 200	74 kDa	Santa Cruz (sc-138065)

### Statistical analysis

All data are presented as mean±SEM. Samples were run in triplicate and each experiment was also run three times. Statistical analyses were performed in Microsoft Office Excel 2017 or GraphPad Prism 7.0c version (Prism, GraphPad Software, San Diego, CA). Gene expression and western blot data were analyzed by Student’s *t*-test or one-way ANOVA with Newman-Keuls multiple comparison test as appropriate; *P* value < 0.05 was considered statistically significant. ^*^p≤0.05, ^**^p≤0.01, ^***^p≤0.001

## RESULTS

### Gene expression of key retinoic acid signaling molecules in Alzheimer’s disease mouse models

**Fig.2:**
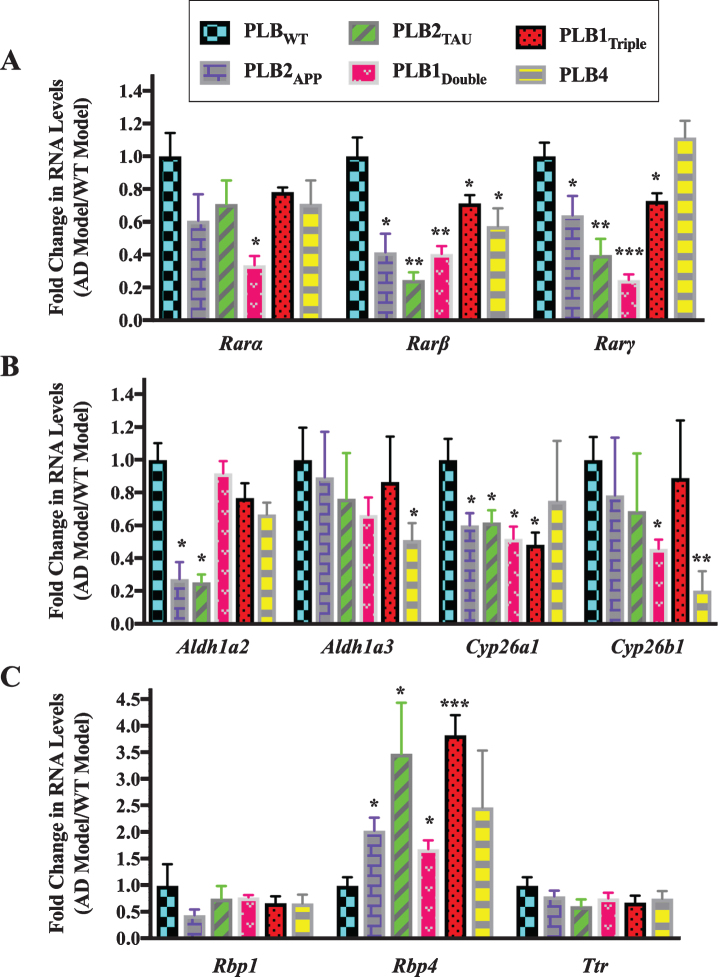
Gene expression analysis of RA signaling system genes in 6-month-old half brains of different AD and FTD transgenic mouse models. RNA was isolated from half brains of AD and FTD mouse models and analyzed by reverse transcription followed by qPCR. A) RA receptors, B) RA enzymes, and C) retinol binding proteins. RNA levels were standardized with respect to the appropriate reference RNA controls in each model and compared to levels in wild type mouse models (WT) which were set at 1. Data represent fold change in the mean of RNA levels with *n*≥6. Error bars indicate standard error of the mean (SEM) (^*^p≤0.5; ^**^p≤0.01; ^***^p≤0.001, one-way ANOVA with Newman-Keuls multiple comparison test).

The expression of RA signaling system genes was screened first in brains from 6-month-old mice of the five types of transgenic mouse models of AD and FTD using a qPCR method to detect RNA levels (Fig. 2). The models used were: PLB2_APP_ knock-in model that expresses mutations in APP [39], PLB2_TAU_ knock-in model that expresses mutations in Tau [40], PLB1_Double_ knock-in model that expresses mutations in APP and Tau, PLB1_Triple_ knock-in model that expresses mutations in APP, Tau, and presenilin 1 [38], and the PLB4 knock-in model that expresses human BACE1 [41]. The number of PLB mice used for gene expression experiments at 6 months of age was 28 PLB_WT_, 6 PLB2_APP_, 8 PLB2_TAU_, 12 PLB1_Double_, 5 PLB1_Triple_, and 6 PLB4.

The data indicated that the RNA levels of 2 subtypes of RARs (β and *γ*) were downregulated in all PLB models except PLB4 compared to the wild type control mice (Fig. 2A). *Rar*β was downregulated by 60% in PLB2_APP_ (*p* = 0.0413) and PLB1_Double_ (*p* = 0.0056), 80% in PLB2_TAU_ (*p* = 0.0013), 30% in PLB1_Triple_ (*p* = 0.0419), and 40% in PLB4 (*p* = 0.0488), while *Rarα* was 66% downregulated only in the PLB1_Double_ model (*p* = 0.0451). *Rarγ* was downregulated in 4 PLB models, with the exception of PLB4, with a decline of 36% in PLB2_APP_ (*p* = 0.0354), 60% in PLB2_TAU_ (*p* = 0.0071), 75% in PLB1_Double_ (*p* = 0.0009), and 27% in PLB1_Triple_ (*p* = 0.0279). The expression of RNA levels of two RA synthesizing enzymes (*Aldh1a2* and *Aldh1a3*) and two RA catabolizing enzymes (*Cyp26a1* and *Cyp26b1*) was also screened in all PLB models (Fig. 2B). *Aldh1a2* was downregulated by 74% in both PLB2_APP_ (*p* = 0.021) and PLB2_TAU_ (*p* = 0.0198), while *Aldh1a3* declined by 50% in PLB4 (*p* = 0.0491). *Cyp26a1* was downregulated by 40% in PLB2_APP_ (*p* = 0.0252) and PLB2_TAU_ (*p* = 0.0249), and 50% in PLB1_Double_ (*p* = 0.0105) and PLB1_Triple_ (*p* = 0.0118). *Cyp26b1* levels declined by 54% in PLB1_Double_ (*p* = 0.0486) and 80% in PLB4 (*p* = 0.0052). The expression of three retinol binding protein genes (*Crbp1*, *Rbp4*, and *Ttr*) was then screened in the PLB models (Fig. 2C). *Rbp4* RNA was upregulated by 103% in PLB2_APP_ (*p* = 0.0263), 248% in PLB2_TAU_ (*p* = 0.0385), 69% in PLB1_Double_ (*p* = 0.0204), and 283% in PLB1_Triple_ (*p* = 0.0007). *Rbp1* and *Ttr* RNA levels did not change in the models.

**Fig.3:**
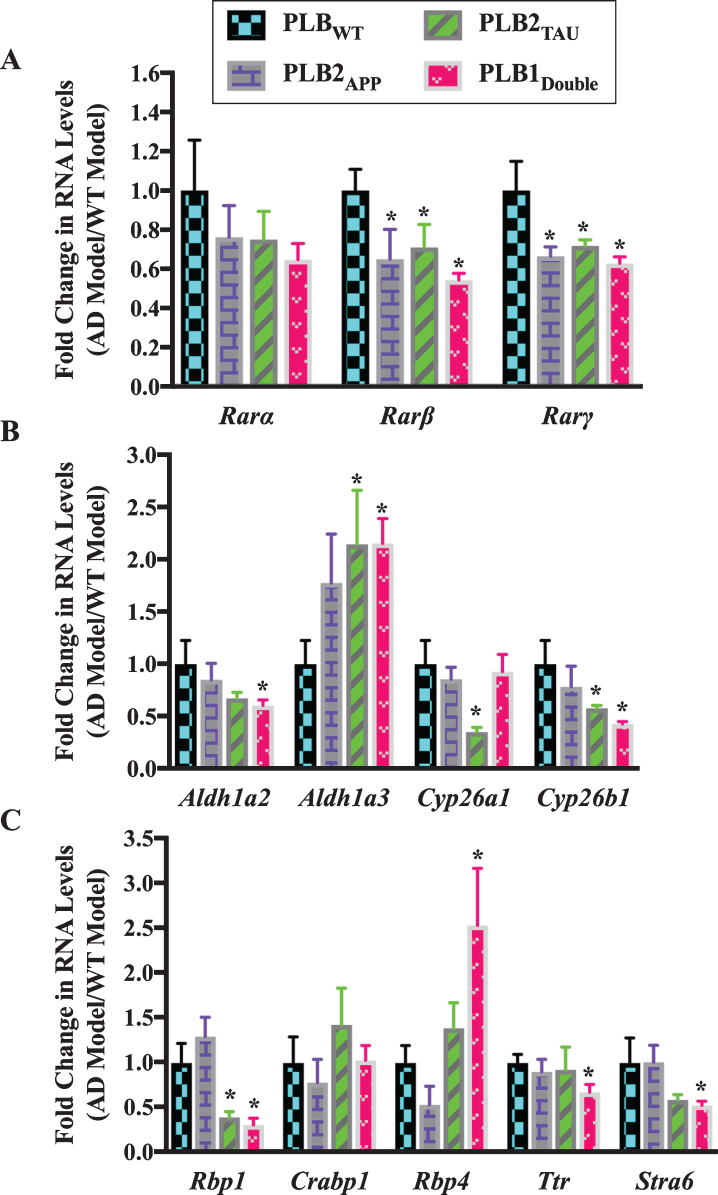
Gene expression analysis of RA signaling system genes in 3-month-old half brains of different AD and FTD transgenic mouse models. RNA was isolated from half brains of AD mouse models and analyzed by reverse transcription followed by qPCR. A) RA receptors, B) RA enzymes, and C) RA binding proteins. RNA levels were standardized with respect to the appropriate reference RNA controls in each model and compared to levels in wild type mouse models (WT) which were set at 1. Data represent fold change in the mean of RNA levels of at least six mouse models. Error bars indicate standard error of the mean (SEM) (^*^p≤0.5, one-way ANOVA with Newman-Keuls multiple comparison test).

Given the qPCR data confirmed that there were abnormalities in the RA signaling system in AD animal models at 6 months of age, the expression of RA related genes was determined at the earlier age of 3 months in PLB2_APP_, PLB2_TAU_, and PLB1_Double_ PLB models (Fig. 3). This tests for genetic changes before significant pathology is evident in the brain of these mice [38, 40]. The number of PLB mice used for gene expression experiments at 3 months of age was 12 PLB_WT_, 4 PLB2_APP_, 12 PLB2_TAU_, and 7 PLB1_Double_.

Analysis of the expression of different RA-related genes indicated that the RA signaling system was also altered in 3-month-old PLB models. The RNA levels of two RAR subtypes (β and *γ*) showed a pattern of downregulation in almost all PLB models, with approximately 35% to 40% decline in *Rar*β and *Rarγ* RNA levels (Fig. 3A). Then, RNA levels of *Aldh1a2*, *Aldh1a3*, *Cyp26a1*, and *Cyp26b1* were screened in PLB models (Fig. 3B). *Aldh1a2* was downregulated by 40% in PLB1_Double_ (*p* = 0.0339). *Cyp26a1* was downregulated by 65% in PLB2_TAU_ (*p* = 0.0361), while *Cyp26b1* was declined by 42% and 57% in PLB2_TAU_ (*p* = 0.0439) and PLB1_Double_ (*p* = 0.0198) lines, respectively. Surprisingly *Aldh1a3* RNA was upregulated by 110% in both PLB2_TAU_ (*p* = 0.0495) and PLB1_Double_ (*p* = 0.0108). The RNA levels of three retinol binding protein genes (*Rbp1*, *Rbp4*, and *Ttr*), a RA binding protein (*Crabp1*) gene and a retinol binding protein receptor gene (*Stra6*) were also screened in the PLB models (Fig. 3C). The RNA level of *Rbp4* was increased by 150% in PLB1_Double_ (*p* = 0.0478) mice. *Stra6* RNA did not change in the PLB2_APP_ model, but it was apparently downregulated in the PLB2_TAU_ (40%) and PLB1_Double_ (50%) models, with a significant decrease evident in the latter (*p* = 0.0468). *Ttr* decreased by 33% in PLB1_Double_ (*p* = 0.0348). *Crabp1* RNA did not change in the PLB models. The *Rbp1* RNA was downregulated by 60% in PLB2_TAU_ (*p* = 0.0495) and 70% in PLB1_Double_ (*p* = 0.0297).

In summary, the expression of a group of RA signaling pathway genes was investigated in 6- and 3-month-old transgenic mice to identify deficits in the RA signaling system. Data indicated that the RNA levels of the RA signaling system were abnormally expressed. RARs, in particular β, *γ*, as well cyp26 enzymes were downregulated. Other genes were more specifically changed, for instance RBP4 RNA levels were upregulated in all tested models at 6 months but did not change at 3 months while RBP1 transcripts decreased only at 3 months.

### Protein expression of key retinoic acid signaling molecules in Alzheimer’s disease mouse models

After investigating the RNA levels of genes encoding the RA signaling system, samples were available to determine RA signaling protein levels in 6-month-half brains of the PLB2_TAU_ and PLB1_Double_ models using the western blot technique. These two models were focused on as those changing most consistently in transcript levels. The number of PLB mice used for protein expression experiments at 6 months of age was 4 PLB_WT_, 5 PLB2_TAU_, and 5 PLB1_Double_.

**Fig.4:**
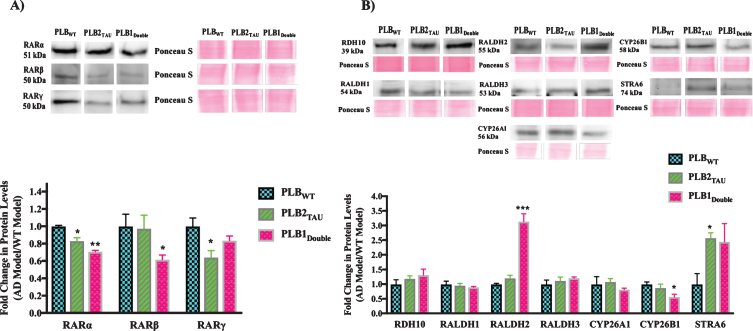
Western blot analysis of RA signaling system proteins in 6-month-half brains of different AD and FTD transgenic mouse models. 50 μg proteins from half brains tissue lysate of PLB mouse models were analyzed by western blot for A) RA receptors or B) RA enzymes and STRA6 proteins and quantified using ImageJ software. Proteins of interest were normalized to total proteins levels measured using Ponceau S staining and compared to protein levels in wild type mouse models (WT) which were set at 1. Shown are mean values of at least four mouse models. Error bars indicate standard error of the mean (SEM) (^*^p≤0.5; ^**^p≤0.01; ^***^p≤0.001, one-way ANOVA with Newman-Keuls multiple comparison test).

The three subtypes of RAR (*α*, β, *γ*) were downregulated in all AD mice models compared to the wild type control mice (Fig. 4A). RAR*α* was downregulated by 17% and 30% in PLB2_TAU_ (*p* = 0.013) and PLB1_Double_ lines (*p* = 0.0037), respectively. RARβ was only downregulated by 40% in the PLB1_Double_ model (*p* = 0.0288). RAR*γ* levels were reduced in PLB2_TAU_ by 36% (*p* = 0.0484). The protein levels of several of the RA synthesizing/degrading enzymes along with RBP4 receptor STRA6 were also screened in the PLB models (Fig. 4B). RDH10 (retinol dehydrogenase enzyme for synthesizing retinal from retinol) did not change in PLB models. RALDH2 and STRA6 proteins were upregulated in some PLB models, with the former being increased by 200% in PLB1_Double_ (*p* = 0.0002) and the latter by 157% in PLB2_TAU_ (*p* = 0.0186). CYP26B1 was downregulated by 43% in PLB1_Double_ (*p* = 0.0295).

In summary, the levels of RA signaling pathway proteins were investigated in 6-month-old transgenic mice to determine if there were any deficits in this system at the protein level. The RARs (*α*, β, *γ*) were the most consistently downregulated, while STRA6 levels were upregulated in all tested models.

### Influence of synthetic retinoids on expression of retinoic acid-related AD genes in mixed neural/glial primary cultures

The decline in RA signaling in varied PLB models supports the concept of this pathway as a target for AD treatment. A new group of ligands for the RARs have been developed called RAR-Ms, initially screened for dual genomic and non-genomic activity [30]. As an initial genomic test of the RAR-Ms to regulate genes in a beneficial way for AD treatment, 17 genes either directly associated with AD, or providing neuroprotection or acting in an anti-inflammatory fashion, were initially tested on rat hippocampal and cortical mixed primary neural/glial cultures. Cultures were treated with a series of RAR-M compounds at 10 nM concentration after 14 DIV for 24 h and RNA levels were measured by the qPCR technique. For each experiment, 3 wells of 6-well plate were used per RAR-Ms treatment, and each experiment was repeated three times.

The effect of RAR-Ms on the RNA levels of amyloid processing genes was first examined in cortical and hippocampal mixed primary cultures. The AD related genes examined for RAR-Ms regulation were: Neprilysin (*Mme*), insulin degrading enzyme (*Ide*), A disintegrin and metalloproteinase domain-containing protein 10 (*Adam10*), amyloid precursor protein (*App*), and β-site amyloid precursor protein cleaving enzyme 1 (*Bace1*). These genes were previously reported to be regulated by RA [24, 51–54].

**Fig.5:**
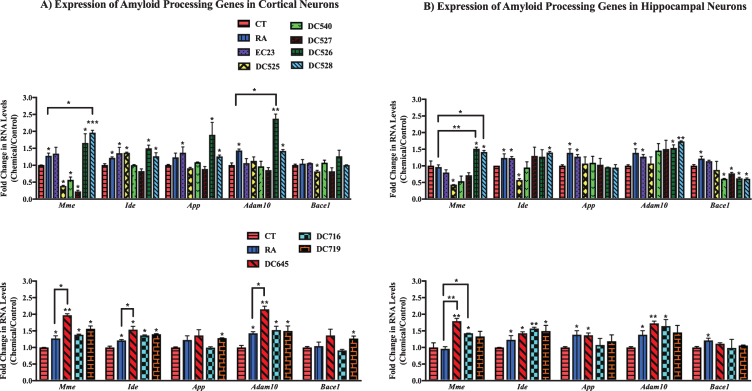
qPCR analysis of amyloid processing genes in primary hippocampal and cortical cultures treated with RAR-Ms. Primary rat cortical (A) and hippocampal (B) cultures were treated with 10 nM RAR-M for 24 h. RNA was isolated and analyzed by reverse transcription followed by qPCR. Amyloid processing gene RNA levels were standardized with respect to the Actb RNA control and compared to levels in control untreated cells (CT) which were set at 1. Shown are mean values of three biological replicates analyzed in triplicate. Error bars indicate standard error of the mean (SEM) (^*^p≤0.05; ^**^p≤0.01, ^***^p≤0.001, one-way ANOVA with Newman-Keuls multiple comparison test).

In cortical cultures (Fig. 5A) the RNA levels of the *Mme* gene were significantly upregulated by DC528 and DC645 compared to both RA and the control, while DC526, DC716, and DC719 increased *Mme* levels significantly compared to the control only. DC525, DC540, and DC527 significantly downregulated the expression of *Mme* gene. *Ide* was upregulated significantly by RA, EC23, DC525, DC526, DC528, DC716, DC719, and DC645. DC645 induction of *Ide* was significant compared to RA as well. *App* was significantly upregulated by EC23, DC526, DC528, and DC719. *Adam10* was upregulated significantly by DC528, DC716, DC719, DC526, and DC645, with the latter two compounds also being significantly greater than RA. *Bace1* was significantly upregulated by DC719 and downregulated by DC525.

In hippocampal cultures (Fig. 5B), *Mme* was significantly upregulated by DC526, DC528, DC645, and DC716 with this increase being significant compared to RA and the control. DC525 treatment significantly downregulated *Mme* levels. *Ide* was significantly upregulated by EC23, DC528, DC645, DC716, and DC719. DC525 treatment downregulated *Ide* gene levels. *App* levels increased significantly with EC23 and DC645 treatments. *Adam10* RNA levels increased in EC23, DC526, DC528, DC645, and DC716 significantly. *Bace1* was downregulated by DC540, DC527, DC526, and DC528.

**Fig.6:**
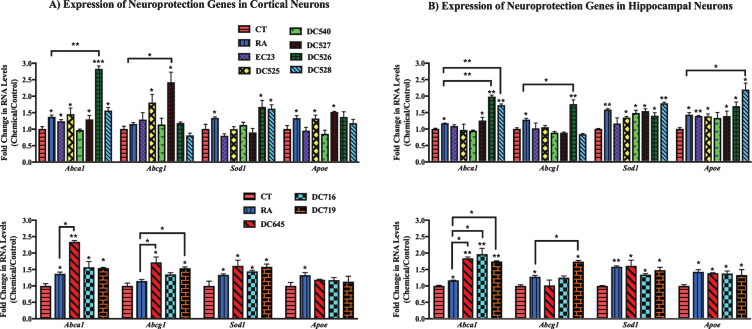
qPCR analysis of neuroprotection genes in primary hippocampal and cortical cultures treated with RAR-Ms. Primary cortical (A) and hippocampal (B) cultures were treated with 10 nM RAR-M for 24 h. RNA was isolated and analyzed by reverse transcription followed by qPCR. RNA levels were standardized with respect to the Actb control and compared to levels in control untreated cells (CT) which were set at 1. Neuroprotective genes were upregulated by RAR-M treatment. Shown are mean values of three biological replicates analyzed in triplicate. Error bars indicate standard error of the mean (SEM) (^*^p≤0.05; ^**^p≤0.01, ^***^p≤0.001 one-way ANOVA with Newman-Keuls multiple comparison test).

In addition, the effect of RAR-Ms on the expression of genes involved in neuroprotection was investigated in rat primary cultures (Fig. 6). The neuroprotection related genes examined for RAR-Ms regulation were: two ATP-binding cassette (ABC) transporters A1 and G1 (*Abca1* and *Abcg1*), the Cu-Zn superoxide dismutase (*Sod1*), and apolipoprotein E (*Apoe*). These genes were previously reported to be regulated by RA [55–57].

From analysis of cortical culture data (Fig. 6A) RNA levels of *Abca1* were significantly upregulated by all RAR-Ms except DC540, while DC526 and DC645 were shown to be more effective than RA. *Abcg1* was upregulated by DC525, DC527, DC645, and DC719, with the latter three compounds being more efficacious than RA. *Sod1* gene was induced to a notable extent by DC526, DC528, DC645, DC716, and DC719, and *Apoe* levels were shown to be increased by treatment with DC525 and DC527 treatments.

In the case of hippocampal cultures (Fig. 6B), *Abca1* was upregulated by DC526, DC528, DC645, DC716, and DC719 compared to control and RA, while DC527 increased *Abca1* compared to control only. *Abcg1* was upregulated significantly by DC526 and DC719 compared to RA and the control. *Sod1* RNA was induced by all RAR-Ms except EC23. All RAR-Ms treatments induced *Apoe*, with DC528 exhibiting particularly effective activity compared to both RA and the control.

The effect of the RAR-Ms on the expression of genes involved in neuroinflammation and neuroprotection was then examined in the primary cultures. The inflammation related genes examined for regulation by the RAR-M compounds were: two C-C motif chemokine ligands, number 3 and 5 (*Ccl3* and *Ccl5*), tumor necrosis factor alpha (*Tnf*), and inducible nitric oxide synthase (*Nos2*) as well as two types of interleukins 12 and 1 beta (*Il-12* and *Il-1*β). In addition, two neuroprotective growth factors were examined, insulin growth factor 1 and 1 (*Igf1* and *Igf2*). These genes have all been reported to be regulated by RA previously [23, 58, 59].

**Fig.7:**
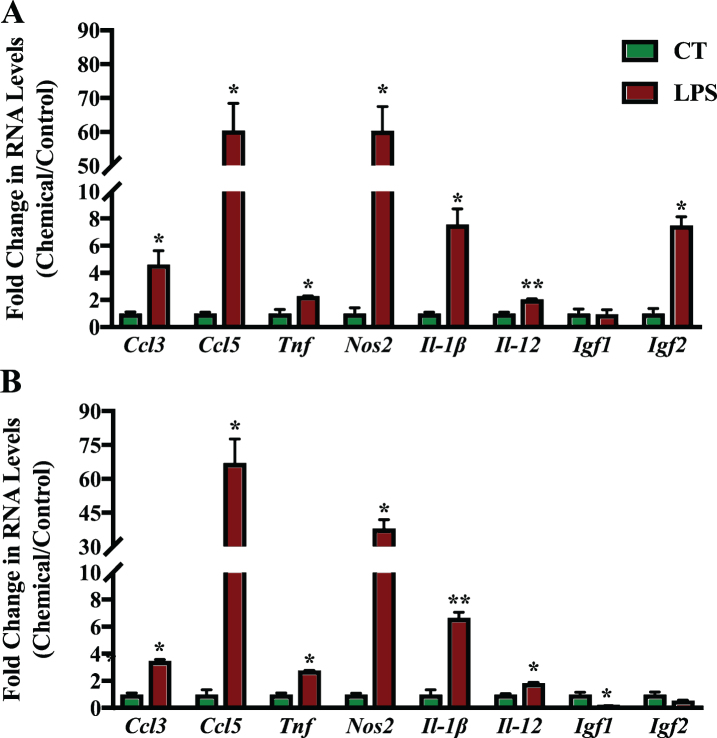
qPCR analysis of inflammation regulated genes in primary hippocampal and cortical cultures treated with LPS. Primary cortical (A) and hippocampal (B) cultures were treated with 1 μg/ml LPS for 6 h to induce inflammation. RNA was isolated and analyzed by reverse transcription followed by qPCR. Inflammatory gene expression was standardized with respect to the Actb RNA control and compared to levels in control untreated cells (CT) which were set at 1. The expression of all genes was upregulated during inflammation except Igf1 which was downregulated significantly in hippocampal cultures. Shown are mean values of three biological replicates analyzed in triplicate. Error bars indicate standard error of the mean (SEM) (^*^p≤0.05; ^**^p≤0.01, student’s *t*-test).

Mixed primary neuronal and glial cultures were first treated with 1 μg/ml LPS for 6 h to induce inflammation (Fig. 7). LPS treatment induced the expression of all inflammation related genes significantly in the cortex cell culture, except for *Igf1*. In hippocampal cultures, all genes were also upregulated except for *Igf1* and *Igf2*. *Igf1* levels were reduced significantly in hippocampal cultures after LPS treatment. *Ccl5* was the gene induced to the greatest extent during inflammation followed by *Nos2* and *Il-1*β.

After the induction of inflammation, the expression of these inflammation related genes was screened in the primary cultures treated with synthetic RAR-Ms to determine how the expression of these genes changed in response to the RAR-M compounds.

**Fig.8:**
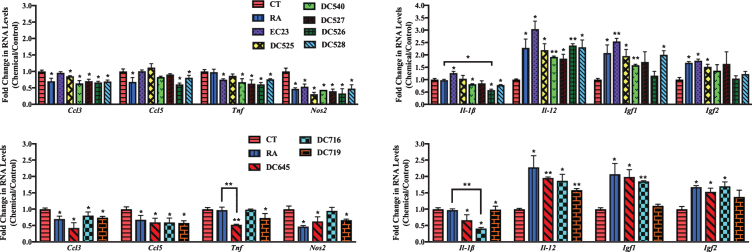
qPCR analysis of inflammation regulated genes in primary cortical cultures treated with LPS followed by RAR-Ms. Primary cortical cultures were treated with 1 μg/ml LPS for 6 h to induce inflammation, and then with 10 nM RAR-M for 24 h. RNA was isolated and analyzed by reverse transcription followed by qPCR. Inflammatory genes were standardized with respect to the Actb RNA control and compared to levels in control untreated cells (CT) which were set at 1. The expression of all genes was downregulated after RAR-M treatment except for Il-12, Igf2, and Igf2 which were upregulated. Shown are mean values of three biological replicates analyzed in triplicate. Error bars indicate standard error of the mean (SEM) (^*^p≤0.05; ^**^p≤0.01, one-way ANOVA with Newman-Keuls multiple comparison test).

In cortical cultures (Fig. 8), all RAR-Ms decreased the RNA levels of the *Ccl3* gene except EC23. *Ccl5* was downregulated by RA, DC526, DC528, DC645, DC716, and DC719 significantly. *Tnf* was downregulated by all RAR-Ms except RA, DC525, and DC716, with DC645 being the most effective. *Nos2* RNA levels were downregulated by all RAR-Ms except DC716. *Il-1*β was notably upregulated by EC23 and, in contrast, downregulated by DC540, DC526, DC528, DC645, DC716, and DC719, with DC526 and DC716 being significantly more effective compared to RA. *Il-12* RNA levels were upregulated by all RAR-Ms. *Igf1* RNA levels were increased significantly by all RAR-Ms except DC527, DC526 and DC719. Treatment with RA, EC23, DC525, DC645, and DC716 caused a significant upregulated in *Igf2* gene expression.

**Fig.9:**
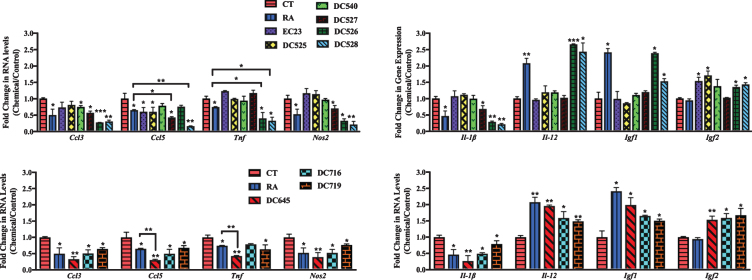
qPCR analysis of inflammation regulated genes in primary hippocampal cultures treated with LPS and RAR-Ms. Primary hippocampal cultures were treated with 1 μg/ml LPS for 6 h to induce inflammation, and then with 10 nM RAR-M for 24 h. RNA was isolated and analyzed by reverse transcription followed by qPCR. Inflammatory genes were standardized with respect to the Actb RNA control and compared to levels in control untreated cells (CT) which were set at 1. The expression of all genes was downregulated after RAR-M treatment except Il-12, Igf2, and Igf2 which were upregulated. Shown are mean values of three biological replicates analyzed in triplicate. Error bars indicate standard error of the mean (SEM) (^*^p≤0.05; ^**^p≤0.01; ^***^p≤0.001, one-way ANOVA with Newman-Keuls multiple comparison test).

In hippocampal cultures (Fig. 9), all RAR-Ms decreased the RNA levels of the *Ccl3* gene except EC23 and DC525. *Ccl5* was downregulated by all RAR-Ms except DC540 and DC526, with DC527, DC528, and DC645 being significantly more effective compared to RA. *Tnf* was downregulated by RA, DC719, DC526, DC528, and DC645, with the latter three compounds being more effective than RA. *Nos2* and *Il-1*β RNA levels were downregulated by RA, DC527, DC526, DC528, DC645, DC716, and DC719. *Il-12* and *Igf1* RNA levels were upregulated by RA, DC526, DC528, DC645, DC716, and DC719. *Igf2* was significantly induced by all RAR-Ms except RA, DC540, and DC527, compared to both control and RA.

In summary, novel RAR-Ms were screened for their ability to regulate the expression of 17 genes involved in either amyloid processing, neuroprotection, or neuroinflammation in rat mixed primary cultures. These initial tests of the therapeutic potential of RAR-Ms indicated that different ligands had varied effects on the investigated genes and DC526, DC528, and DC645 were more effective than RA in regulating almost all genes in a therapeutically beneficial way. Other RAR-Ms had beneficial effects, but on a limited number of genes and not all of them such as DC540 and DC527 while RAR-Ms such as DC525 regulated some genes in the opposite direction to most ligands.

## DISCUSSION

It is well recognized that the complex pathology of dementias such as AD means it cannot be adequately studied in a single animal model, as they are largely based on a single hypothesis, and usually rely on rare genetic mutations detected in AD (APP, BACE, presenilin) or FTD (tau) patients. There is also a need to examine the contribution of individual genes versus bi- or trigenic models of AD [60]. Several studies have investigated the changes in retinoid signaling in over-expression models, but none have so far investigated genetically comparable models [61], expressing mutated tau and/or APP as well as BACE1, in knock-in models. It has here been demonstrated that endogenous RA signaling changes differentially in five different transgenic PLB mouse lines, at the gene and/or protein level. Genes encoding components of the RA signaling system were abnormally expressed in these mouse models at 6 months of age. Further, analysis of gene expression in 3-month-old mice illustrated early deficits in RA signaling, such as the reduction of *Rar*β and *Rarγ* receptors in all PLB models and the decline in *Cyp26b1* and *Rbp1* in both PLB2_TAU_ and PLB1_Double_ models. This is indicative that such changes may be an initiator event in AD.

The three subtypes of RA receptors (*α*, β, *γ*) were downregulated in the PLB models at both RNA and protein levels and this decrease will lead to an almost inevitable fall in the strength of RA signal in the brain. The link between vitamin A and dementia had been made by Corcoran and colleagues who reported that *Rarα* was downregulated in the forebrain of 6-month-old vitamin A deficient rats accompanied by a loss of choline acetyltransferase (ChAT) and followed by Aβ deposition in 1-year-old VAD rats [19]. It was also reported that *Rar*β and/or *Rxrγ* null mice exhibit impairment in LTP and working memory [7] and that there is an approximately 30% decline in *Rar*β and *Rxr*β/*γ* expression in the hippocampus of aged mice [62]. Furthermore, pathological samples from AD patients showed *Rarα* deficits along with Aβ deposition in the surviving neurons in the meningeal vessels of the neocortex [19]. These results imply that a decline in RA receptors may be among the events initiating AD.

The initial catalyst resulting in an early decrease in expression of the RA receptors is unknown. One possibility is that this is an indicator of an early decline in RA levels in the brain. All RARs are inducible by RA [63, 64] and in particular RAR *α* and β in the CNS [19] can decline when RA levels fall. A clue to what triggers the fall in RA may lie in the role microglia have in brain RA metabolism. Activation of microglia increases their capacity to degrade RA [65], possibly part of the inflammatory response to AD, while Aβ is able to inhibit RA synthesis, potentially blocking microglia RA synthetic capacity [24]. It is of note that decline in RAR expression and RA signaling is also evident in the aging mouse and that this is reversible by RA treatment [66]. Although the cognitive decline in AD and normal aging each has its distinguishable features, there is also significant overlap between the two in genetic susceptibility and epigenetic input [67, 68]. Epigenetic modification has been proposed in the treatment of both AD [69] and aging [70]. This may be another mechanism by which RA can influence cognitive decline and aging given the capacity of RA to regulate epigenetic change [71].

Changes in other RA signaling components, enzymes, and binding proteins were more complex in the PLB models suggesting that they may be secondary to the decline in the RA receptors or secondary to other events in the AD brain. The *Aldh1a2* gene encoding the enzyme that synthesizes RA was downregulated at the RNA level in 6-month-old PLB2_APP_ and PLB2_TAU_ PLB models and 3-month-old PLB1_Double_ PLB model, but surprisingly it was upregulated at the protein level at 6 months. The increase in RALDH2 protein could be a feedback/compensatory mechanism at the translational level in order to increase RA synthesis in response to the decline of RA signal. A similar phenomenon was described in the neuroblastoma cell line LA-N-5, where RALDH was increased when the cells were depleted in RA and was restored to normal levels on addition of RA [72]. It is of particular note that there is a two-fold increase in the RALDH protein in the hippocampus and parietal cortex of AD patients [72]. Further, an increase in RALDH2 protein was reported in the spinal cord of rats during inflammation [73, 74], and one of the features of AD is inflammation. This may be a key interacting event leading to the increase of RALDH2 in the AD mouse models, regulating at a translational level despite the decrease in *Aldh1a2* transcript. It has been reported in a number of studies that mRNA and protein expression levels do not always correlate [75, 76] and this is also the case for the retinoid signaling system in the adult brain [77]. Several factors could influence the mRNA-protein correlation such as posttranscriptional and posttranslational modifications, mRNA and protein stability, protein half-life and the lower rate of transcription compared to translation in mammalian cells.

The RA catabolizing enzymes (CYP26A1 and CYP26B1) were both downregulated at the RNA and protein levels in all our PLB models. Both these genes are reliant on RA for induction as a regulatory mechanism to protect against excess RA and their expression falls when RA is low [78–81]. Their decline in the PLB mice may reflect the overall reduction in RA signaling in the AD/FTD brain.

RBP4 is a liver secreted retinol carrier protein in the circulation [82]. Its function in the brain is unknown but an increase in RBP4 protein levels was reported in the brain of APP/PSEN1 AD model mice [83], and it is upregulated in the liver and forebrain of 8-month-old PLB4 mice [84]. The current study reproduced these findings and *Rbp4* gene levels were upregulated in the brains of all 6-month-old PLB models but at 3 months only in the PLB1_Double_ mouse line. High levels of RBP4 protein have been found in the amyloid plaques of human AD patients [85]. Particularly intriguing is the correlation of decreasing RBP4 in the CSF with progression of normal to mild cognitive impairment to AD, suggesting a movement of RBP4 between brain compartments with disease, although this study was with a small patient pool [86]. Changes in RBP4 may be part of the metabolic disorder linked with AD [87] and the inconsistent change between mouse models at 3 months in RBP4 transcript suggests that this may not be an early change in disease.

Alterations in STRA6, the RBP4 cell surface receptor [88], may also be part of these metabolic disruptions in AD. STRA6 changed in a complex fashion, being downregulated at the RNA level but upregulated at the protein level in PLB2_TAU_ and PLB1_Double_ AD mouse models (Fig. 4). STRA6 acts as a cell surface signaling receptor activating the JAK2/STAT5 signaling transduction pathway following RBP4/retinol binding which induces the expression of suppressor of cytokine signaling 3 (SOCS3) protein, which in turn inhibits insulin signaling [89]. CRBP1 is part of the STRA6 complex on the intracellular side and assists with transport of retinol into the cell and sites of its use [88], and is also linked to metabolic disorders [90]. *Rbp1* gene expression levels were downregulated in 3-month-old PLB2_TAU_ and PLB1_Double_ PLB models. Thus, the overexpression of STRA6 along with the upregulated RBP4 levels in the brain and downregulated CRBP1 on the intracellular side of the receptor might jointly interrupt movement of retinol into cells and dysregulate metabolism in AD. Furthermore, TTR, a carrier protein for retinol and thyroxine in the plasma and CSF [91], was decreased in 3-month-old PLB1_Double_ at the gene level. Lower levels of TTR protein were reported previously in the CSF of AD patients [92].

The animals used are refined AD models with low expression of AD-related genes and the studies presented here on prodromal stages of disease add to the accumulating evidence that RA signaling declines early in neurodegeneration leading to the proposal of the use of RAR ligands as therapeutics to boost RA signaling [93]. There is currently preliminary pilot data on the use of retinoids in clinical trials, but the potential therapeutic effects of retinoids in AD are still at an early stage of investigation. For example, the results of the clinical trial of bexarotene (RXR agonist) suggests that it may reduce brain amyloid and increases serum Aβ_1–42_ in ApoE4 noncarriers in mild to moderate AD patients [94]. Moreover, acitretin (RAR agonist) entered Phase II clinical trials in Germany in 2010, and the preliminary results reported 25% increase in AβPPs-*α* in CSF of mild to moderate AD patients in the treatment group [29].

In this study, we have provided an initial test of a new RAR-M class of retinoid, selected by screening for both genomic and non-genomic activity, the drugs were tested on primary neuron/glia mixed cultures from hippocampus and cortex. The RAR-Ms tested herein are significantly more chemically stable than RA, which can be easily oxidized and isomerized in the presence of light, oxidants, and excessive heat [95, 96]. The RAR-M family is exemplified by EC23 [33] which has been shown to be 8-fold greater in potency (EC_50_) than RA in genomic activity (activation of a retinoic acid response element), while exhibiting similar non-genomic (activation of ERK kinase) potency (EC_50_) to RA [30].

Several studies had already shown the involvement of RA signaling in amyloid pathology. RA deficiency leads to Aβ accumulation in the cerebral cortex and blood vessels [18, 19] and a group of amyloid processing proteins is upregulated by RA, including BACE1 [51], APP [52], ADAM10 [53], IDE, and NEP [24, 53]. Induction of these proteins by RA will be therapeutically advantageous, with the conspicuous exception of BACE1 in which induction could potentially worsen the disease. In this new study, RA, along with a group of synthetic RAR-M compounds, upregulated the amyloid processing genes. Of the tested compounds, RAR-Ms DC526, DC528, and DC645 were more effective at inducing *Mme, Ide,* and *Adam10* in both cortical and primary neurons compared to control, with no or weak induction or downregulation of Bace1. DC528 and DC645 induction of *Mme* was notably stronger compared to RA in both cortical and primary neurons. DC526 and DC645 increased *Adam10* in cortical neurons compared to RA. It was also notable that although these RAR-Ms were selected according to their potent genomic and non-genomic biological activities, there was significant variance in the genes they activated; for instance, DC525 reduced the levels of genes such as *Mme,* which is disadvantageous in the treatment of AD.

RA has neuroprotective properties since it lowers cholesterol levels by upregulating the expression of genes involved in cholesterol homeostasis, such as Abca1 and Abcg1 [56]. These ABC cholesterol transporters play a role in the lipidation of ApoE, the protein involved in the transport of cholesterol to neurons via low density lipoprotein receptors, which promotes the clearance of Aβ [97–99]. The RAR-M compounds were shown to upregulate the expression of *Abca1*, *Abcg1*, and *Apoe* involved in cholesterol regulation and neuroprotection, with DC526, DC528, and DC645 being significantly stronger compared to RA in most cultures. Regulation of cholesterol homeostasis is important for synaptic transmission and neural plasticity [100], and excess cholesterol levels promote the generation of Aβ [101].

Other neuroprotective genes investigated included *Sod1*, the encoded protein known to bind copper and zinc ions and quench free superoxide radical species in the body. There are elevated levels of oxidative stress in AD patients and transgenic mouse models of AD [102, 103] and AβPP and Aβ impair mitochondrial import channels and electron transport chain, leading to the generation of free reactive oxygen species [104]. *Sod1* was notably upregulated by DC526, DC528, DC645, and DC716 compared to the control in both cortical and hippocampal cultures.

The ability of the RAR-Ms to suppress an LPS induced inflammatory reaction was also investigated in the mixed neuronal/glial cultures, adding the RAR-M after inflammation was induced for 6 hours. Neuroinflammation has been proposed to play a role in AD and elevation in inflammatory response and mediators is associated with cognitive decline and loss of neurons in AD patients and transgenic animal models of AD [105, 106]. Microglia act to clear Aβ through phagocytosis; however, this interaction activates signaling cascades which, in turn, mediate the release of different pro-inflammatory cytokines (TNF*α*, IL-1β, etc.), chemokines (CCL3, CCL5, etc.), and reactive oxygen/nitrogen species which further promotes Aβ plaques [107, 108]. Each of the RAR-Ms downregulated the expression of multiple genes involved in inflammation whether in cortical or hippocampal cultures such as *Ccl3*, *Ccl5*, *Tnf*, *Nos2*, and *Il-1*β, with DC526, DC528, and DC645 being significantly more effective compared to RA in decreasing these inflammatory markers in most cultures. Surprisingly though, RAR-Ms upregulated *Il-12* gene expression. It was reported previously that IL-12 may have anti-inflammatory activity [109]. Moreover, the regulation of this interleukin might depend on choosing the right treatment time, as it can fall and then rise following the induction of inflammation [110].

LPS induced inflammation may also promote neurodegeneration through control of the expression of growth factors. In hippocampal cultures, LPS repressed both *Igf1* and *Igf2* (although markedly inducing *Igf2* in cortical cultures reminiscent of the effect of LPS on human microglia [111]). IGF1 and IGF2 modulate the survival of neurons and protect against cytokine mediated death of neurons in human neuronal cultures [111]. IGF1 increases *α*-secretase processing of AβPP and decreases Aβ levels [112]. IGF1 and IGF2 have both been proposed as treatments for AD [113–115]. Several of the RAR-Ms upregulated the expression of both *Igf1* and *Igf2* genes and by increasing endogenous levels of the growth factors, this circumvents the major problem of IGF1 or IGF2 treatment—poor BBB permeability. Small lipophilic ligands for RARs generally cross the BBB [28, 116], while RAR ligands like Am80 [117] and acitretin [118] have demonstrable therapeutic action in AD mouse models, indicative of their capacity to cross the BBB.

Overall, this study strengthens the argument of decreased RA signaling in early AD and the subsequent dysregulation of the RA signaling system, which could contribute to the underlying pathogenesis. The earliest consistent change in all models examined was in RA receptors at both RNA and protein level, and these may be earliest changes following the onset of tau and amyloid dysregulation. The many other alterations in genes involved in RA signaling may be secondary and in part compensatory. For instance, the change in RALDH2 may, like CYP26A1 and CYP26B1, be a result of feedback regulation of the RA signaling system, or alternatively may be a response to inflammation. Changes in RBP4, STRA6 and CRBP1, associated with vitamin A’s role in metabolic disease [87] may be linked with the metabolic abnormalities that occur in AD [119].

The initial test of the therapeutic potential of RAR-Ms found that DC526, DC528, and DC645 had a strong anti-inflammatory effect downregulating most inflammation genes more effectively than RA. They would be expected to have neuroprotective activity as they induced the expression of both *Igf1* and *Sod1*. DC526, DC528, and DC645 regulate AβPP processing in a beneficial way, inducing *Mme* and *Adam10* to a greater extent than RA and also inducing *Ide*, but having a lower effect on *Bace1* than RA. Several other RAR-Ms also had beneficial effects on a more limited number of genes – these were more potent than RA but did not have the broader positive effects of DC526, DC528, and DC645. For example, DC540 and DC527 effectively repressed inflammatory genes but also downregulated *Mme* and *Ide* which are important for degrading Aβ. Such RAR-Ms may have future potential to be used to target specific pathways.

Of the ligands for RA receptors investigated as AD therapeutics the most interest has been directed towards bexarotene, an agonist of retinoid X receptors (RXR) which are related to the RARs but with a wider range of action because they heterodimerize with many nuclear receptors including PPAR, thyroid hormone receptor, vitamin D receptors and others, including RAR. After the initial promise [55], not all initial findings were replicated [120] but continued research suggests that bexarotene may still have benefit as a therapeutic [121]. An important route of action is the clearance of Aβ by upregulating ApoE levels [55]. Of the RAR-Ms investigated herein, a few significantly upregulated *Apoe* levels, such as DC525 in cortical neurons and DC528 in hippocampal neurons. However, there are a number of adverse effects including cardiovascular risk due to hypertriglyceridemia [122]. In comparison, the specificity of the RAR-Ms for a single nuclear receptor should reduce side-effects and the variety of therapeutic actions of these drugs to be anti-inflammatory, neuroprotective, and promote the non-amyloidogenic pathway, suggesting promise in the future for treatment of AD and other neurodegenerative diseases.

## Supplementary Material

Supplementary Figures
