# Supplementary material for: Decay in Retinoic Acid Signaling in Varied Models of Alzheimer’s Disease and In-Vitro Test of Novel Retinoic Acid Receptor Ligands (RAR-Ms) to Regulate Protective Genes
Source: J Alzheimers Dis. 2020 Feb 4;73(3):935–54. doi: 10.3233/JAD-190931 (PMC7081102; doi:10.3233/JAD-190931)

**Supplementary Material**

**Decay in Retinoic Acid Signaling in Varied Models of Alzheimer’s Disease and In-Vitro Test of Novel Receptor Acid Receptor Ligands (RAR-Ms) to Regulate Protective Genes**

**Supplementary Figure 1.** qPCR analysis of RA signaling genes in 6-month-old half brains of the PLB1Double transgenic mouse model. RNA was isolated from half brains of A) 6 wildtype and 7 mutant male and B) 4 wildtype and 5 mutant female PLB1Double mice and analyzed by reverse transcription followed by qPCR. RNA levels were standardized with respect to Gapdh and Ywhaz reference RNA controls and compared to levels in wild type mouse models (WT) which were set at 1. There was no change in the expression of RA signaling system genes between male and female mice. Data represent fold change in the mean of RNA levels. Error bars indicate standard error of the mean (SEM) (*p≤0.5, **p≤0.01 student’s t test).


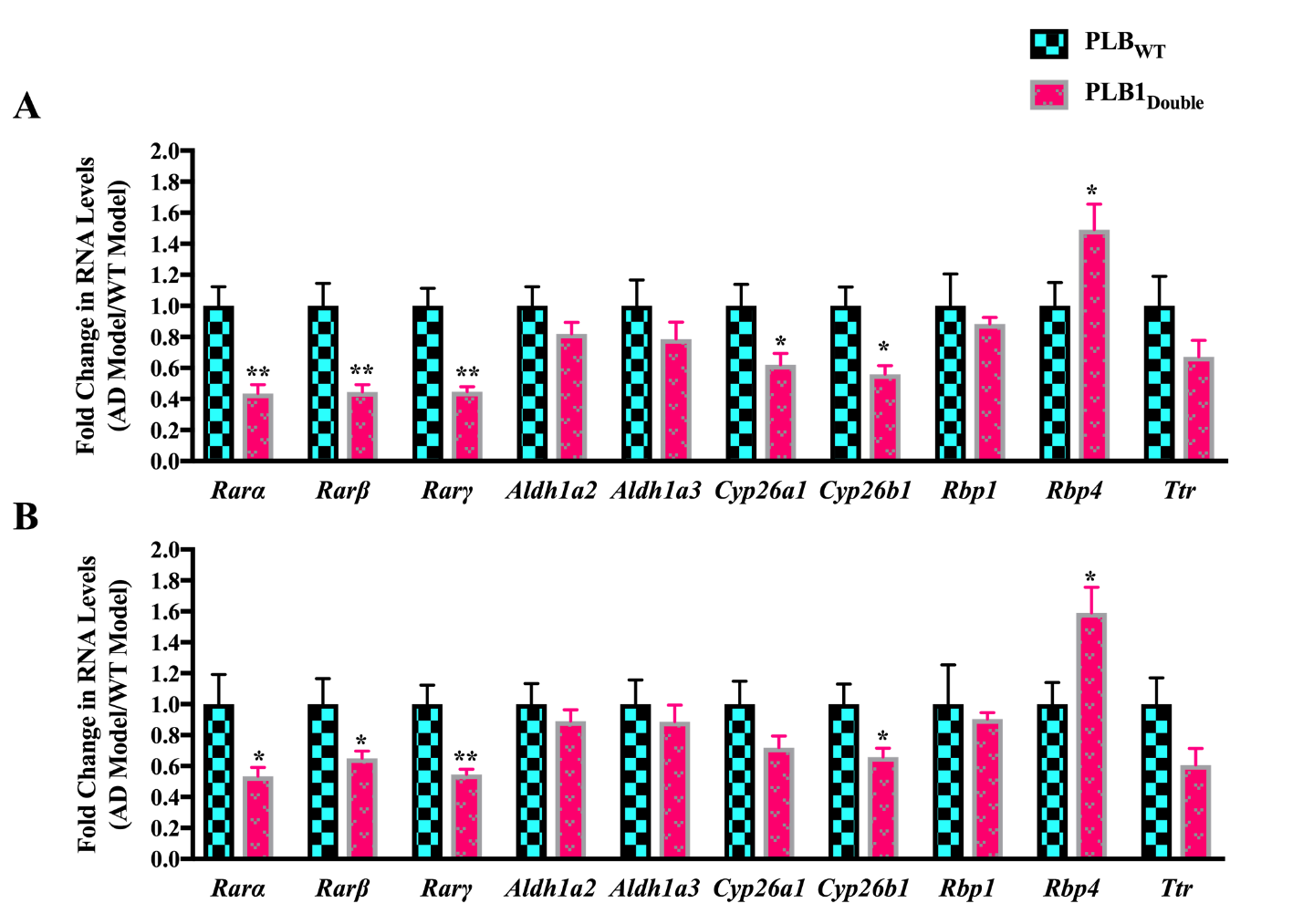


**Supplementary Figure 2.** qPCR analysis of amyloid processing genes in primary hippocampal and cortical cultures treated with RA for 6 and 24 h. Primary hippocampal and cortical cultures were treated with RA for either A) 6 h or B) 24 h. Then RNA was isolated and amyloid processing genes RNA and as control Actb RNA levels were analyzed by reverse transcription followed by qPCR. Amyloid processing genes RNA levels were standardized with respect to the Actb RNA control and compared to levels in control untreated cells (CT) which were set at 1. Shown are mean values of three biological replicates analyzed in triplicate. Error bars indicate standard error of the mean (SEM) (*p≤0.05, student’s t-test). Data showed that 6 h was not enough to induce a change in RNA levels after RA treatment especially in hippocampal cultures compared to 24 h. Amyloid processing genes were upregulated by RA treatment.


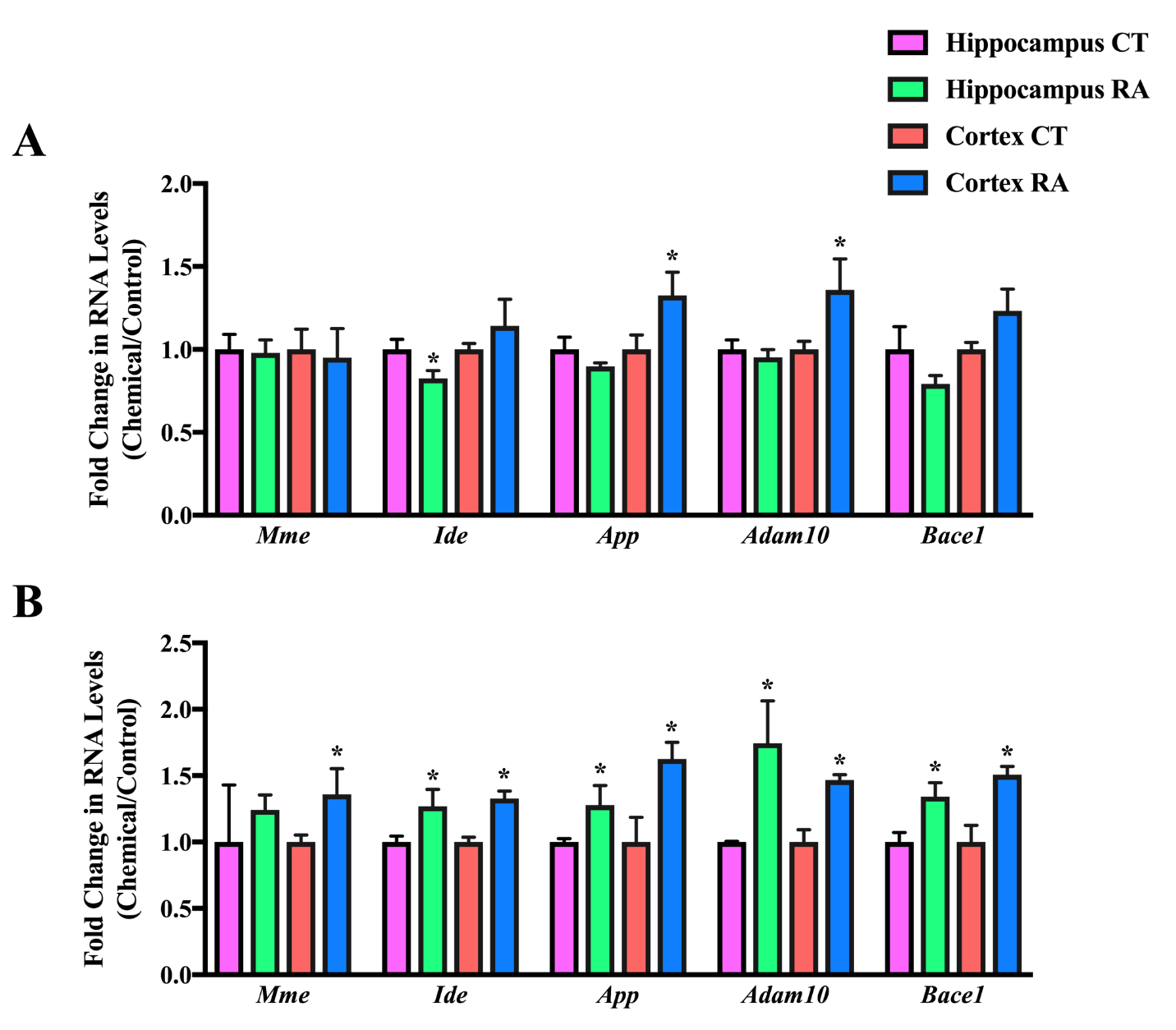

Supplement: Supplementary Figures [file jad-73-jad190931-s001.docx]
